# Amyloid Goiter: A Peruvian Case Series

**DOI:** 10.17925/EE.2024.20.2.16

**Published:** 2024-04-02

**Authors:** José Paz-Ibarra, Marcio Concepción-Zavaleta, Juan Eduardo Quiroz-Aldave, José Somocurcio-Peralta, María Belén Tite Haro, Paola Solis-Pazmino

**Affiliations:** 1. Department of Medicine, School of Medicine, Universidad Nacional Mayor de San Marcos, Lima, Peru; 2. Division of Endocrinology, Hospital Nacional Edgardo Rebagliati Martins, Lima, Perú; 3. CaTaLiNA: Cancer de tiroides en Latinoamerica, Quito, Ecuador; 4. Universidad Científica del Sur, Lima, Perú; 5. Division of Non-communicable diseases, Endocrinology research line, Hospital de Apoyo Chepén, Chepén, Perú; 6. Department of Pathological Anatomy, Hospital Nacional Edgardo Rebagliati Martins, Lima, Perú; 7. Universidad San Francisco de Quito, Quito, Ecuador; 8. Surgery Department, Santa Casa de Misericórdia in Porto Alegre, Porto Alegre, Brazil

**Keywords:** Amyloidosis, chronic renal insufficiency, goiter, nodular goiter, Peru, thyroid diseases

## Abstract

**Introduction**: Amyloid goiter (AG) is a rare cause of thyroid swelling, characterized by deposits of amyloid protein in the thyroid tissue. It can be associated with primary or secondary amyloidosis. Its prevalence in multinodular goiter cases is 0.17%, with rare clinical detection before surgery. **Case series:** This Peruvian case series comprised three female patients and one male patient, with ages ranging from 28 to 65 years. All individuals had pre-existing inflammatory diseases and reported symptoms including dyspnoea, dysphagia and dysphonia. Upon physical examination, all patients exhibited a grade III goiter. Fine-needle aspiration reported colloid goiter. Three out of the four patients underwent total thyroidectomy and histochemistry revealed AG with positive Congo red staining. **Discussion:** AG is an uncommon clinical entity. It has been reported to occur more frequently in males, with an average age of diagnosis of 40 years. In our series, we observed a broad age range of patients receiving diagnoses, spanning from 28 to 65 years, with a predominance in females. The consideration of AG should be extended to every patient with an underlying chronic systemic inflammatory disease, especially end stage renal disease. In this context, AG should be included in the differential diagnosis for patients with multinodular goiter exhibiting progressive growth and causing compressive symptoms at the cervical level without affecting thyroid function, as demonstrated in our series. **Conclusion:** AG, a rare condition, warrants suspicion in the presence of a giant goiter with an underlying systemic inflammatory disease.

Amyloid goiter (AG) is a benign condition characterized by the deposition of amorphous proteinaceous material in the thyroid gland to an extent that results in detectable enlargement during clinical evaluation.^1–3^ Amyloid can infiltrate the thyroid gland in 15–50% of individuals with primary amyloidosis (known as “amyloid light chain” or AL amyloidosis) and in 20–80% of patients with secondary amyloidosis (“amyloid A” or AA amyloidosis).^1,3,4^ However, clinically significant enlargement resulting from amyloid deposition is exceptionally rare.^1^ The prevalence of AG among cases of multinodular goiter has been found to be 0.17%.^2^ The nature of the goiter results in compression symptoms such as dysphagia, dyspnoea and hoarseness.^5^

A definitive diagnosis is established through histopathological examination following thyroid surgery.^1,6^ Subsequently, immunochemical staining is employed to ascertain the fibril type, utilizing specific antibodies to distinguish the type of amyloidosis. In AL amyloidosis, the primary fibrillar protein is amyloid L, whereas in AA amyloidosis, the fibril subunit is amyloid A.^2^ The hypothesis regarding AG due to AA amyloidosis physiopathology is included in Figure 1.^7,8^

In this manuscript, we present a series of cases involving four patients. A summary of their key characteristics is provided in*Table 1*.

## Case series

### Case 1

A 28-year-old man with a medical history of pulmonary tuberculosis, bronchiectasis and end-stage renal disease (ESRD) on haemodialysis (HD) due to renal amyloidosis presented to the surgery department with a complaint of a 3-year history of painless neck swelling that had been increasing in size, along with dyspnoea, dysphagia and dysphonia. Physical examination revealed a grade III goiter without skin changes, bruit or retrosternal extension (Figure 2).

Thyroid function tests were normal. Neck ultrasound revealed an enlarged thyroid gland with hyperechogenicity of the parenchyma. The size of the right lobe was 130 x 70 x 50 mm, and the left was 100 x 60 x 40 mm. Fine-needle aspiration (FNA) reported colloid goiter (Bethesda II) with numerous adipose tissue fragments.

The patient underwent total thyroidectomy, and the histopathological examination revealed thyroid amyloidosis with positive Congo red staining (Figure 3). He has been followed up for 5 years using hormonal reposition treatment.

**Figure 1:**
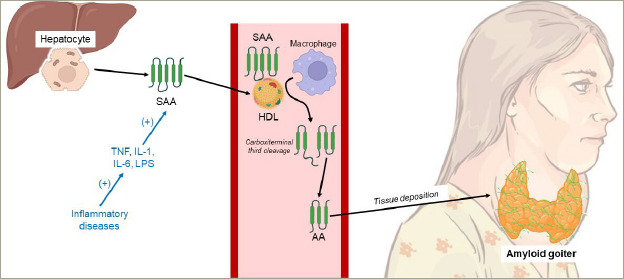
Pathophysiology of AA amyloidosis^7,8^

*Hepatocytes generate SAA protein under the influence of TNF, IL-1, IL-6, and LPS. The resulting SAA molecules bind to plasma HDL and undergo proteolytic processing, with a significant cleavage taking place at position 76, leading to the release of the carboxyterminal third of the molecule, thereby forming AA. Inflammatory diseases are associated with an augmented synthesis of SAA due to heightened levels of proinflammatory cytokines. Consequently, AA accumulates in the form of cross-β-sheet fibrillar deposits. The factors determining the specific sites of deposition for these fibrils remain unidentified*

*AA = amyloid A; HDL = high-density lipoprotein; IL-1 = interleukin-1; IL-6 = interleukin-6; LPS = lipopolysaccharide; SAA = serum amyloid A; TNF = tumor necrosis factor.*

### Case 2

A 65-year-old woman with a medical history of rheumatoid arthritis, Sjögren syndrome and ESRD due to nephroangiosclerosis and polycystic kidney disease presented to the surgery department with a complaint of a 6-year history of painless neck swelling that had been increasing in size, along with dyspnoea and compression symptoms. On physical examination, she presented with a grade III goiter (Figure 4).

Thyroid function tests were normal. Neck ultrasound revealed an enlarged thyroid gland with hyperechogenicity of the parenchyma. The size of the right lobe was 40 x 30 x 40 mm, and the left was 60 x 40 x 30 mm. FNA reported colloid goiter (Bethesda II).

The patient underwent total thyroidectomy, and the histopathological examination revealed thyroid amyloidosis with positive Congo red staining (Figure 5). She had 3 years of follow-up and used hormonal reposition treatment. Unfortunately, she died due to complications in haemodialysis.

**Table 1:** Key features of the patients included in this case series

Feature	Case 1	Case 2	Case 3	Case 4
**Age (years)**	28	65	67	43
**Sex**	Male	Female	Female	Female
**Medical history**	Pulmonary TB, BCH, ESRD on HD	RA, Sjögren syndrome, ESRD	RA, HTN, osteoporosis, ESRD on HD	DILD, BCH, aspergillosis, RF, NS
**Illness time (years)**	3	6	2	1
**Presentation**	Size-i ncreasing, painless neck swelling	Size-i ncreasing, painless neck swelling	Size-i ncreasing, painless neck swelling	Cervical pain
Compressive symptoms*	Yes	Yes	Yes	Yes
**Physical evaluation**	Grade III goiter	Grade III goiter	Grade III goiter	Grade III goiter
**Thyroid function**	Normal	Normal	Normal	Normal
**Imaging†**	Enlarged thyroid, parenchymal hyperechogenicity	Enlarged thyroid, parenchymal hyperechogenicity	Multinodular goiter	Enlarged thyroid, parenchymal hyperechogenicity
**Thyroid size**	RL: 130 x 70 x 50 mm LL: 100 x 60 x 40 mm	RL: 40 x 30 x 40 mm LL: 60 x 40 x 30 mm	RL: 80 x 40 x 40 mm LL: 70 x 50 x 40 mm	RL: 80 x 40 x 40 mm LL: 70 x 50 x 35 mm
**Presurgical FNA biopsy**	Bethesda II goiter with adipose tissue fragments	Bethesda II goiter	Bethesda II goiter with scant, oncocytic-appearance cells	Thyroid amyloidosis and Congo red (+)
**Surgery**	Total thyroidectomy	Total thyroidectomy	Total thyroidectomy	No surgery due to a high surgical risk
**Pathology**	Thyroid amyloidosis and Congo red (+)	Thyroid amyloidosis and Congo red (+)	Thyroid amyloidosis and Congo red (+)	Not applicable
**Follow-up (years)**	5	3	1	In course
**Treatment**	LT4, calcium, and calcitriol	LT4, calcium, and calcitriol	LT4, calcium, and calcitriol	Palliative

*Compressive symptoms: dyspnoea, dysphagia and dysphonia.

*†Imaging: ultrasound or computed tomography.*

*BCH = bronchiectasis; DILD = diffuse interstitial lung disease; ESRD = end-stage renal disease; FNA = fine-needle aspiration; HD = haemodialysis; HTN = hypertension; LL = left lobe;*

*LT4 = levothyroxine; NS = nephrotic syndrome; RA = rheumatoid arthritis; RF = respiratory failure; RL = right lobe; TB = tuberculosis.*

**Figure 2:**
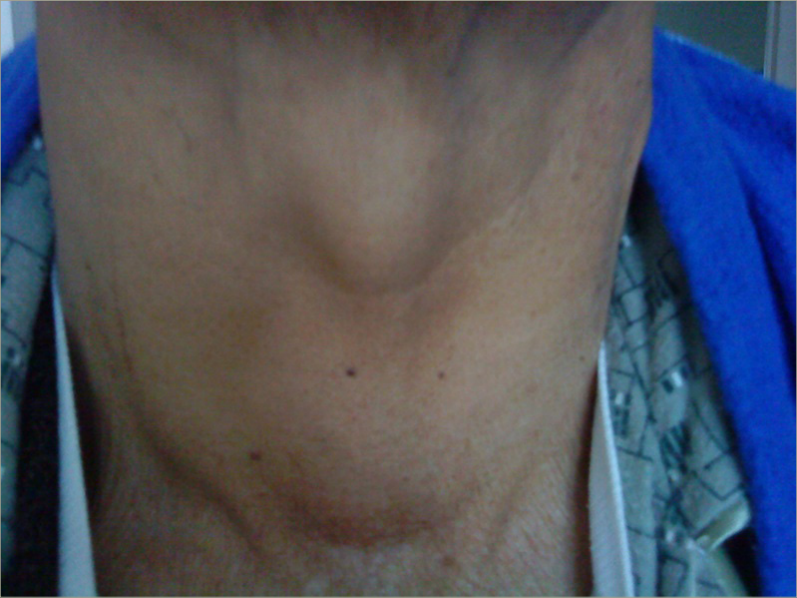
Gross examination of goiter in patient 1

*Giant, multinodular, 3 years of evolution*

### Case 3

A 67-year-old female with a medical history of rheumatoid arthritis, arterial hypertension, osteoporosis and ESRD on HD presented to the surgery department with a complaint of a 2-year history of painless neck swelling that had increased in size, along with dyspnoea and dysphagia. On physical examination, she presented with a grade III goiter.

Thyroid function tests were normal. Computed tomography showed multinodular goiter. The right lobe was 80 x 40 x 40 mm, and the left was 70 x 50 x 40 mm (Figure 6). FNA-reported goiter (Bethesda II) with scant cellularity composed of cells with an oncocytic appearance with large nuclei and some with the nucleolus, scant colloid.

The patient underwent total thyroidectomy, and the histopathological examination revealed thyroid amyloidosis with positive Congo red staining (Figure 7). She had a 1-year follow-up using hormonal reposition treatment.

### Case 4

A 43-year-old female with a medical history of diffuse interstitial lung disease, bronchiectasis, aspergillosis, respiratory failure and nephrotic syndrome due to renal amyloidosis presented with a 1-year history of cervical pain, dyspnoea and dysphonia. On physical examination, she presented with a grade III goiter.

**Figure 3:**
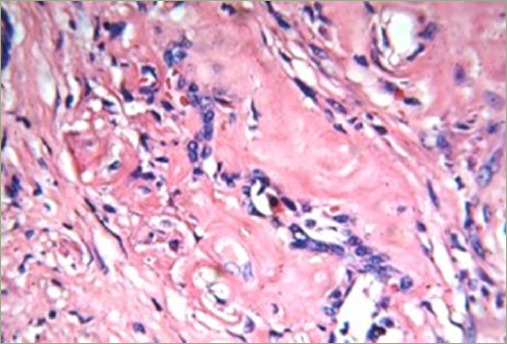
Thyroid amyloidosis hystopathology in patient 1

*Congo red (+) (HE 40X)*

**Figure 4:**
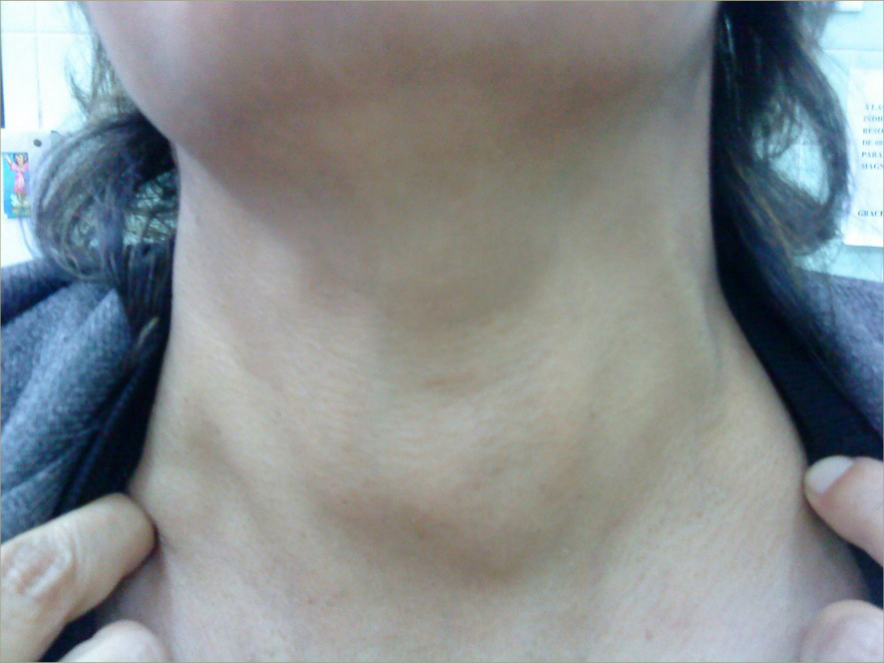
Macroscopic view of goiter in patient 2

*Grade III, multinodular, 6 years of evolution*

Thyroid function tests were normal. Neck ultrasound revealed an enlarged thyroid gland with hyperechogenicity of the parenchyma. The size of the right lobe was 80 x 40 x 40 mm, and the left was 70 x 50 x 35 mm. FNA biopsy revealed thyroid amyloidosis, confirmed by positive Congo red staining. The patient did not undergo thyroidectomy due to a high surgical risk, and received palliative treatment.

## Discussion

A systematic review found that the mean age of patients with AG is 43.7 years, with ages ranging from 23 to 75 years.^3^ This aligns with our observations in the four reported cases. However, it was noted that two-thirds of the patients in the review were male, which contrasts with the results of our case series, where the majority of the cases (3 patients) were female.^3^

**Figure 5:**
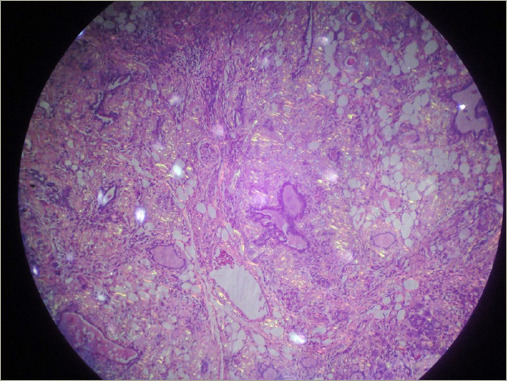
Thyroid amyloidosis hystopathology in patient 2

*In vision under polarized light with Congo red (+)*

**Figure 6:**
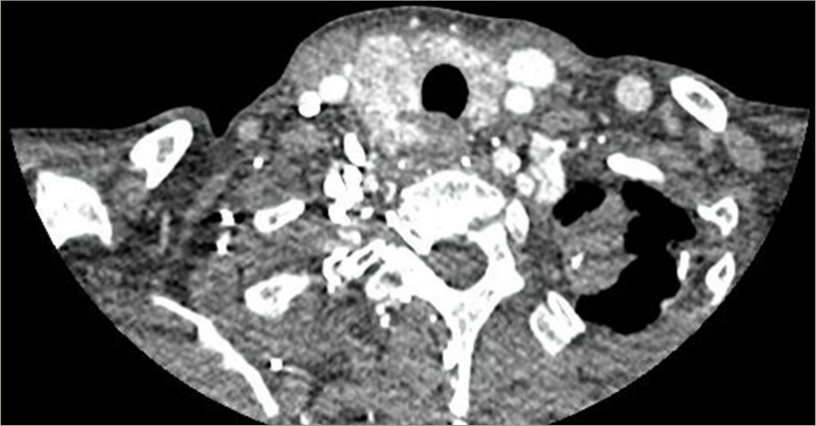
Computed tomography of the neck, case 3

*RTL: 80 x 40 x 40 mm; LTL: 70 x 50 x 35 mm; Itsmus: 35 x 20 x 20 mm. It shows a multinodular goiter with a right predominance of 2 years of evolution and progressive growth LTL = left thyroid lobe; RTL = right thyroid lobe (instead of LTD).*

While AG is not associated with predisposing comorbidities in AL amyloidosis, in AA systemic amyloidosis, it has been found to be associated with chronic inflammatory diseases, including chronic infections, familial Mediterranean fever, inflammatory bowel disease (especially Crohn’s disease), rheumatoid arthritis, bronchiectasis and chronic kidney disease.^1,2,9^ AA amyloidosis is the most common form in developing countries.^1^

Seventy percent of patients with AG presented with a painless, rapidly enlarging neck mass over weeks to years, mirroring the observations in all our cases.^3^ One-third of these patients experienced upper airway compressive symptoms such as dyspnoea, dysphonia and dysphagia, which were also evident in all four of our cases.^3^ In this context, malignancy, especially anaplastic thyroid cancer and non-Hodgkin malignant lymphoma, should be ruled out, as they are known to cause rapid enlargement of the thyroid and compressive symptoms.^1,3^ It is noteworthy, however, that unlike malignant thyroid tumors, which typically present unilaterally, AG affects the thyroid in a bilateral and diffuse manner.^3^

Regarding goiter, the World Health Organization classifies it into stage 0 (no goiter), stage IA (palpable but not visible goiter), stage IB (palpable goiter but visible only when the neck is fully extended), stage II (goiter easily visible with the neck in a normal position) and stage III (very large goiter).^10^ The distribution of patients with AG is 30% in stage 0, 20% in stage IA, 30% in stage II, and 20% in stage III. All our cases were in stage III.^3^

Ultrasound examination usually reveals diffuse enlargement of the thyroid gland, with amyloid deposition detected as complex or hypoechoic masses.^3^ However, in some cases, the thyroid gland may exhibit hyperintense areas or present a nodular appearance.^3^ Two of our patients underwent ultrasonography, showing enlargement of the thyroid gland with parenchymal hyperechogenicity. One patient underwent computed tomography, revealing a multinodular goiter; however, this imaging technique is not routinely performed. Laboratory studies showed normal thyroid function in 80% of cases, similar to all of our cases.^3^

**Figure 7:**
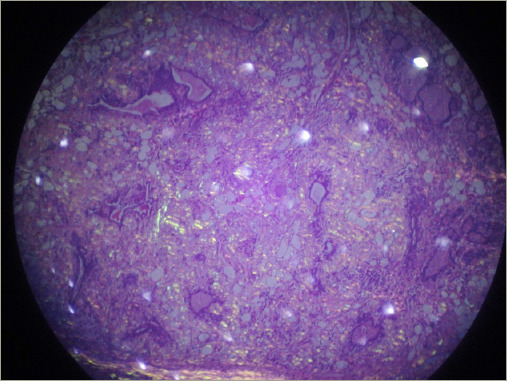
Thyroid amyloidosis hystopathology in patient 3

*In vision under polarized light with Congo red (+)*

AG is defined as the clinical enlargement of the gland due to the presence of extracellular amyloid-l ike material, displaying distinctive Congo red staining and apple-green birefringence when observed under polarized light microscopy.^3^ All our patients demonstrated this phenomenon in FNA cytology. Around 25% of FNAs conducted on AG may only reveal atypical follicular cells, limiting its ability to differentiate between AG and medullary thyroid carcinoma.^1,11–13^ Additionally, in our case series, thyroid FNA reported colloid goiter in three of our four patients. This way, achieving an accurate diagnosis of AG typically requires histopathological examination of resected surgical specimens.^1^ The thyroid gland typically appears enlarged, and the thyroid parenchyma is characterized by multiple inhomogeneous nodules, ranging from firm to soft gelatinous consistency. Some cases may present with simple cysts, while others exhibit haemorrhagic ones. The cut surface varies in colour, ranging from grey-white to light brown or pale yellow.^3,6^ Regarding medullary thyroid carcinoma, it usually manifests as a cervical mass, causing compressive symptoms. FNA biopsy may reveal variable histological features, including salt-and-pepper chromatin, multinucleation, and solid nests of plasmacytoid or spindled cells in a fibrous stroma. Calcitonin and carcinoembryonic antigen are essential for diagnosis.^14^

Surgical management is primarily preferred to alleviate local symptoms (cervical compression, asphyxia, dysphagia) caused by the glands increased volume.^2^ Total thyroidectomy is the preferred treatment, although subtotal thyroidectomy can be considered.^3,15^ All our patients underwent total thyroidectomy.

## Limitations

The reported case series limitation is the inability to classify the type of amyloid; therefore, based on the clinical context, it is assumed that all cases are instances of secondary amyloidosis. Additionally, we lack images for case 4.

## Conclusion

AG is an uncommon disease. The development of a giant goiter in the presence of an underlying systemic inflammatory disease should raise suspicion of amyloidosis. FNA can provide valuable guidance for diagnosis and treatment. However, a definitive diagnosis is typically achieved through immunohistochemical analysis following total thyroidectomy.
